# Gone with the Protocol: Searching for unpublished documents from unregistered clinical trials

**DOI:** 10.1192/j.eurpsy.2025.2191

**Published:** 2025-08-26

**Authors:** G. Tomei, F. Visconti, I. A. Cristea

**Affiliations:** 1 Department of General Psychology, University of Padova, Padova, Italy

## Abstract

**Introduction:**

Trial protocols and manuals are critical documents outlining core elements of a clinical trial, such as theoretical background, recruitment strategies, intervention structure and components, and control comparators. Availability of these documents is essential as it prevents trial duplication, helps detect or avoid research misconduct, and facilitates tracking of trial results. Moreover, a detailed description of intervention components is crucial for the methodological rigor of advanced meta-research approaches, such as component network meta-analysis (cNMA). However, clinical trials often fail to make these documents publicly accessible, either before or after the trial ends, for instance as supplementary materials to publications. Attempts to obtain these documents by contacting researchers directly are frequently unsuccessful. Therefore, alternative avenues for retrieving these materials must be explored.

**Objectives:**

This study aims to assess the feasibility of retrieving trial protocols and manuals through alternative sources, such as ethics committees/IRBs, funding bodies, and sponsors.

**Methods:**

In the context of a cNMA on psychotherapeutic interventions for eating disorders, we identified 34 published studies (12 on anorexia nervosa and 22 on bulimia nervosa); for each, we reviewed the full-text publications to identify potential sources to retrieve trial protocols and manuals. To this end, we adopted a systematic, stepped approach, investigating: 1) explicit mentions of published trial protocols; 2) trial registration details, including ethics committees/IRBs; 3) institutions or clinics whose ethics committees approved the protocol; 4) specific trial sites or study sponsors/funders; 5) first author’s affiliation.

**Results:**

Of the 34 publications analyzed: 1) 2 studies had published trial protocols; 2) 11 studies were registered in trial databases and reported ethics committee or IRB details; 3)16 studies identified the ethics committee or IRB that approved the protocol; 4) 26 studies reported one or more specific trial sites or funding bodies/sponsors; 5) 31 studies provided the first author’s institutional affiliation.

Overall, 26 studies had at least one contact associated with organizations that would have reviewed the trial documents, and 31 studies had at least one institutional contact potentially connected to the trial’s documentation. For 3 studies no information on ethics committees, trial site, or author affiliation was available.

**Conclusions:**

Despite the recognized importance of making trial protocols publicly available, spontaneous dissemination remains rare in clinical trials on psychotherapeutic interventions. In the absence of published protocols or trial registration details, our feasibility study highlights that full-text publications of trial results can offer multiple potential points of contact that may provide access to such documentation.

**Disclosure of Interest:**

None Declared

